# Assessment of a block curriculum design on medical postgraduates’ perception towards biostatistics: a cohort study

**DOI:** 10.1186/s12909-018-1232-0

**Published:** 2018-06-19

**Authors:** Chen Li, Ling Wang, Yuhai Zhang, Chanjuan Li, Yongyong Xu, Lei Shang, Jielai Xia

**Affiliations:** Department of Health Statistics, School of Preventive Medicine, Fourth Military Medical University, Changlexilu Road #169, Xi’an, Shaanxi 710032 People’s Republic of China

**Keywords:** Biostatistics, Medical education, Perceptions, Curriculum design

## Abstract

**Background:**

Biostatistics is a key but challenging subject in medical curricula that is usually delivered via a didactic approach in China. However, whether it is the best teaching approach to improve the learner’s competency, especially for medical postgraduates is yet to be proved. Therefore, a block curriculum design was initially developed to provide selective education to the postgraduates towards the professional career of their interest. A questionnaire was designed to assess the students’ perceptions toward biostatistics as these affective factors might impact the learning process. Thus, the present study aimed to detect whether the new block curriculum design could promote the students’ positive perceptions and further improve the course achievement.

**Methods:**

This cohort study investigated and assessed the perceptions toward biostatistics of the first-year postgraduates undergoing traditional teaching and block teaching, respectively. Structural equation modeling was applied to explore the association between perception and course achievement in the block teaching group.

**Results:**

With a response rate of 97.84 and 96.67% from the two cohorts respectively, 499 block teaching postgraduates had more positive perceptions as compared to 465 traditionally teaching postgraduates with Likert 5-point agreement response mean of 3.50 vs. 3.31 for course value, 3.66 vs. 2.97 for course comment, and 4.29 vs. 4.10 for expectation. Moreover, block teaching students presented superior confidence about academic statistical knowledge, and therefore, 77.96% of them approved of the new teaching approach. Age, specialty, research experience, logical thinking capacity, mathematical basics, and computer basics might influence the postgraduates’ self-assessment ability (all *P* < 0.05). Structural equation modeling confirmed a positive correlation between perceptions and the course achievements with a reasonable fit.

**Conclusions:**

The block curriculum design in the biostatistics course improved the postgraduates’ positive perception and may have had a positive role in improving postgraduates’ achievement in learning biostatistics.

**Electronic supplementary material:**

The online version of this article (10.1186/s12909-018-1232-0) contains supplementary material, which is available to authorized users.

## Background

The concurrent emphasis on evidence-based care prompt the medical professionals to apply statistical tools for providing quality care, which requires an expert level of understanding the biostatistics for study design, data analysis, and result interpretation [1]. Therefore, teaching biostatistics is advocated during the formal training of medical student in all categories [2].

Although biostatistics is well recognized in medical curricula in both developed and developing countries, it is generally considered as a challenging course for teaching and learning [3]. It involves probability theory, mathematics, and computer technology, requiring a high level of logical thinking ability [4]. Compared to the learning methods in other medical curricula via images and memorization, such as anatomy [1], it is rather challenging for students to adapt to the learning methods for biostatistics that which requires a different style of thinking [5]. In addition, medical students often lack in research experience, pushing them to feel confused in understanding statistics without practical experience [6], as the curriculum usually helps the students to acquire statistical knowledge, but does not equip them for learning in a medical environment applicatively .

In the last 30 years, several researchers and faculties have made efforts to reform the teaching modality in order to improve the teaching efficacy to satisfy the different needs of the postgraduate students [7]. For example, the Australian method of teaching statistics focused on problem-based learning may guide other countries [8]. Researchers from England developed a multi-disciplinary approach to teaching medical statistics, which resulted in superior outcomes regarding learning and understanding statistics [9]. The Chinese educators applied blended learning, which combined a modular object-oriented dynamic learning environment for improved outcomes in students’ knowledge, attitudes, and practices [10]. The case-discussion methods utilized by the American educators also garnered success in mastering the material and positive assessment from students [11].

Although much attention has been paid to improve the cognitive aspects of instruction, some affective factors that can influence the learner’s achievement in biostatistics, especially the perception toward biostatistics continue to persist [12, 13]. The perception represents the emotional feelings during the learning process and students’ self-assessment ability including knowledge, skills, and attitudes [14]. Students’ judgments on the interest, importance, and usefulness of the course might influence their learning process and their willingness to engage with the subject. In addition, the judgment of their capability to perform academic tasks may exert an impact on their efforts and academic achievement [15, 16]. Several studies have highlighted the significance of students’ perceptions in contribution towards their academic achievement [17, 18]. Zhang et al. demonstrated significant correlations between course achievement and perceptions toward statistics [19]. Khan et al. showed that high level of motivation for further training of statistical techniques exhibited a satisfactory level of course achievement [20]. Artino et al. found that enjoyment critically affected the subsequent achievement outcomes in medical school and suggested that medical educators should explicitly address the students’ achievement-related emotions [21]. A meta-analysis reported the incremental contribution of psychosocial factors and perceptions in predicting college outcomes [22]. Another meta-analysis also confirmed that achievement in statistics was markedly correlated with attitude component [23].

In China, biostatistics at the postgraduate medical level was primarily delivered by a didactic approach. Students often complained about their anxiousness and confusion during the biostatistics learning process [19]. However, only a few studies have focused on the teaching reform to reduce students’ negative perceptions in the learning process. In this study, a block curriculum design was initially proposed with three blocks representing three different difficulty levels and research directions to ease the learning difficulty and improve the students’ real-life data analysis and hands-on, active learning. A questionnaire for medical postgraduates’ perceptions toward biostatistics was developed to investigate two cohorts of postgraduate students: receiving traditional teaching and block teaching. The study compared the perceptions between the two cohorts and assessed whether the block design could contribute to reducing the students’ negative perceptions during learning biostatistics. This study also explored the association between the students’ perceptions and course achievements. Taken together, we aimed to investigate the students’ positive perceptions under the block teaching situation and further improve their course achievement.

## Methods

### Block curriculum design

As shown in Table 1, traditional biostatistics courses for Chinese medical postgraduates consisted of 60–70 lessons during the first semester, and each lesson was delivered by a tutor with a 30–40 min PowerPoint presentation. The curriculum encompassed descriptive statistics, some key probability distribution concepts, some methods of parameter estimation, hypothesis testing [*t*-test, analysis of variance (ANOVA), chi-square test], nonparametric statistics, and linear correlation and regression. In block teaching design, the curriculum was structured by three blocks. (1) Initially, a basic compulsory block that consisted of 40 lessons including descriptive statistics and some basic parametric and nonparametric statistics. (2) Then one of the three intermediate modules, containing 20 lessons, was provided, that could be selected by postgraduates based on their professional needs. (3) The three intermediate modules were mainly focused on statistical design and analysis for experimental study, observational study, and clinical trial, which provided knowledge about the study design, randomization, sample size estimation, and multivariate statistical analysis. Students who completed the compulsory basic and intermediate blocks would receive credits and could opt for advanced blocks, such as survival analysis and professional statistical software. The teaching methods were not only didactic as the conventional style but blended with a variety of modalities, such as lecture, problem-based learning, massive open online course, and inquiry-based learning. Students were divided into small groups (20 students/group) to undertake a literature review, participate in group discussion, undergo lab practice of the software, and undertake research design outside of the classroom. Therefore, the block curriculum was still 60 lessons for each student, but it was highly focused on coping with their professional needs.

**Table 1:** Comparison in curriculum design between the traditional teaching and the block teaching

Traditional teaching(60–70 lessons)	Block teaching(70 lessons)		
	Basic block(40 lessons)	Intermediate block(20 lessons)	Advanced block(10 lessons)
Study design	Descriptive Statistics	**Module I: Experimental study**	Survival analysis
Descriptive Statistics	Probability distribution	Experimental study design	Questionnaire design and evaluation
Probability distribution	Sampling error	Randomization	Psychological measurement
Sampling error	Hypothesis testing	Sample size estimation	Advanced software practice: SigmaPlot
Parameter estimation	*t*-test	Analysis of variance	Advanced software practice: Origin
Hypothesis testing	Chi-square test	**Module II: Observational study**	
*t*-test	Non-parametric statistics	Observational study design	
Analysis of variance	Linear correlation and regression	Sampling survey	
Chi-square test	Statistical table and chart	Multivariate linear regression	
Non-parametric statistics		Logistic regression	
Linear correlation and regression		**Module III: Clinical trial**	
Statistical table and chart		Clinical trial design	
		Bias	
		Analysis of variance	

### Questionnaire

The questionnaire design group included biostatistics educators, professors involved in the questionnaire design, a psychologist, and postgraduates. Reviewing standard scales and self-designed questionnaires concerning perceptions toward statistics [21, 24–27], and a 5-point Likert-type questionnaire was adopted: 1 = Strongly disagree to 5 = Strongly agree. Ninety first-year postgraduate students were randomly selected to complete the pilot/draft in order to assess the questionnaire’s comprehension, ensure face validity, and provide comments.

The resulting questionnaire (Additional file 1) was structured with 24 items in five sections: (A) value, (B) comment, (C) expectation, (D) reform, and (E) ability. The course value section was concerned with the students’ perceptions of usefulness, difficulty, and worth of biostatistics in their academic career. The course comment section addressed the students’ feelings and comments during the learning process. The expectation section investigated their opinions on teaching measures that might be beneficial to the learning process. Additionally, postgraduates receiving block teaching were required to complete a reform section concerning perception towards the block curriculum design. Finally, an ability section incorporated nine statistical academic questions to survey the students’ self-assessment of knowledge levels that was represented by scores as follows: 1 = never heard, 2 = heard but lacked application, 3 = applied but unaware of rationale, 4 = applied with rationale, and 5 = applied with rationale and software practice. The score of each section was defined as the mean score of items constituted under each section. Higher scores indicated positive perceptions. In addition, a few non-Likert-type items were embedded to elucidate the specific supplementary views. Participants were also requested to provide demographic information with respect to age, gender, specialty, the background of logical thinking ability, mathematical basics, computer basics, and research experience.

### Settings and participants

The study was conducted at the Fourth Military Medical University, China, which has a long history of imparting formal education in biostatistics. Staff and students originated from a wide variety of cultural backgrounds encompassing all medical categories.

Next, we assessed the perception towards biostatistics from two cohorts of postgraduates experiencing different teaching styles. Before the block design was conducted, we investigated the first-year postgraduates’ perceptions, who received the traditional didactic teaching in 2013. After the block teaching reform in 2014, another survey was conducted on the first-year postgraduates who received block teaching in 2015. Finally, two cohorts were compared to assess the impact of the block approach on students’ perception towards biostatistics. The Ethics Committee of the Fourth Military Medical University carefully considered and approved the project proposal. A written informed consent was obtained from all participants before enrolment in the study.

### Procedures

All the first-year postgraduates from 2013 and 2015 were invited to participate in the investigation after completion of the biostatistics curriculum. One week before the final exam, all participants were requested to complete the questionnaire individually in an in-class situation without discussion or collaboration. The students were assured that their answers would not impact their academic achievement or future learning process. No names were registered, and only a control number was allocated that was known only to the principal investigator, who could track the final examination score of the student over the semester.

### Statistical analysis

Data were analyzed using SAS 9.2 (SAS Institute Inc., USA). Descriptive statistics were applied to present the characteristics of the two cohorts. Chi-square test revealed the baseline balance between the two groups, and *t*-test and ANOVA were used to determine the participants’ characteristics associated with mean scores related to perceptions. Multivariable linear regression analysis identified the effect of teaching approaches on perceptions with the imbalance in characteristics between the two groups as covariates. Structural equation modeling (SEM) [28], using M-plus (Muthen & Muthen, Mplus, Version 7), was applied to test the linear relations between latent perceptions and students’ academic achievement. A two-tailed *P*-value of 0.05 was considered as statistically significant with a 95% confidence interval (CI).

## Results

A total of 510 postgraduate students receiving block teaching and 481 receiving traditional teaching participated in the survey. Finally, 499 and 465 participants from both cohorts completed the questionnaire, giving response rates of 97.84 and 96.67%, respectively. Since the students were not required to answer all the questions, unanswered questions were considered as missing data.

### Sample characteristics

The characteristics of the respondents were summarized in Table 2. The distribution of age in both groups ranged from 21.28–47.13 years for block teaching students and 21.04–43.00 years for traditionally teaching students. No significant difference was observed in gender or degree with respect to the response rate. Block teaching students majored less frequently in a clinical career (68.74% vs. 76.34%, *P* < 0.01), but more frequently in medical research (18.64% vs. 16.34%, *P* < 0.01). At least 70% of the participants in both groups reported good or neutral logical thinking ability, mathematical and computer basics, which indicated that students held a basic knowledge and computer operating ability for learning biostatistics. Moreover, block teaching students had more research experience concerning biostatistics than traditional teaching students (61.73% vs. 40.00%, *P* < 0.01).

**Table 2:** Demographic characteristic of participants with traditional teaching and block teaching

Characteristic	Traditional teaching	Block teaching	*P* value
	Number of participants(%)	Number of participants(%)	
	*N* = 465	*N* = 499	
Age			
< 25	218 (46.88)	142 (28.40)	< 0.01
≥ 25	247 (53.12)	357 (71.60)	
Gender			
Male	256 (55.05)	298 (59.72)	0.14
Female	209 (44.95)	201 (40.28)	
Degree			
Academic	321 (69.03)	342 (68.54)	0.87
Professional	144 (30.97)	157 (31.46)	
Specialty			
Clinical	355 (76.34)	343 (68.74)	< 0.01
Research	76 (16.34)	93 (18.64)	
Others	34 (7.31)	63 (12.63)	
Logical thinking ability			
Very poor	9 (1.94)	6 (1.20)	0.23
Poor	30 (6.45)	29 (5.81)	
Neutral	249 (53.55)	247 (49.50)	
Good	159 (34.19)	184 (36.87)	
Very good	18 (3.87)	33 (6.61)	
Mathemetical basic			
Very poor	18 (3.87)	14 (2.81)	0.69
Poor	83 (17.85)	94 (18.84)	
Neutral	254 (54.62)	283 (56.71)	
Good	96 (20.65)	98 (19.64)	
Very good	14 (3.01)	10 (2.00)	
Computer basics			
Very poor	15 (3.23)	16 (3.21)	0.02
Poor	94 (20.22)	80 (16.03)	
Neutral	285 (61.29)	287 (57.52)	
Good	60 (12.90)	104 (20.84)	
Very good	11 (2.37)	12 (2.40)	
Research experience			
No	279 (60.00)	199 (38.27)	< 0.01
Yes	186 (40.00)	300 (61.73)	

### Perceptions and attitudes

The cumulative percentage of variance was assessed as 0.76. The internal consistency reliability estimates for each section were shown in Table 3. All reliability estimates were within the desired range with Cronbach’s α coefficient 0.70–0.72 [29], except for that in the reform section.

**Table 3:** Mean score of perceptions towards biostatistics for postgraduates receiving traditional teaching and block teaching^a^

Survey sections	Mean Score (SD) ^a^		Mean Score Difference (B-T)	Partial regreesion Coefficient (95%CI)^b^	*P* value^c^
	Traditional Teaching	Block Teaching			
Section A: Value(0.71^b^)	3.31 (0.50)	3.50 (0.56)	0.19	0.14 (0.07,0.20)	< 0.01
A1. I am interested in biostatistics	3.48 (0.72)	3.68 (0.76)	0.20		
A2. I have no obstacle in learning biostatistics	2.79 (0.82)	2.88 (0.84)	0.09		
A3. I gained useful statistical knowledge and skills by taking this course	3.71 (0.64)	3.91 (0.65)	0.25		
A4. I know how to use statistical knowledge in my professional career	3.28 (0.70)	3.48 (0.75)	0.20		
Section B: Comment(0.74 ^b^)	2.97 (0.55)	3.66 (0.58)	0.69	0.67 (0.59,0.74)	< 0.01
B1. This course introduced adequate knowledge to satisfy my practical career goals	2.66 (0.85)	3.46 (0.82)	0.80		
B2. The course framework is scientific and reasonable	3.28 (0.83)	3.50 (0.85)	0.22		
B3. The theory and practice are well combined in the teaching modality	2.65 (0.94)	3.87 (0.71)	1.22		
B4. The course content is appropriate	3.29 (0.71)	3.80 (0.72)	0.51		
Section C: Expectation(0.70 ^b^)	4.01 (0.47)	4.29 (0.46)	0.28	0.25 (0.18,0.31)	< 0.01
C1. Need more software practice lessons	3.97 (0.62)	4.07 (0.73)	0.10		
C2. Need more practical workshop for research design and analysis in real study	4.09 (0.60)	4.39 (0.58)	0.30		
C3. Provide comprehensive guide handbook for biostatistics like other medical subjects	4.01 (0.77)	4.46 (0.59)	0.45		
C4. Give public lecture introducing the latest and hot issues relating to biostatistics	4.02 (0.63)	4.25 (0.65)	0.23		
Section D: Reform(0.45 ^b^)		3.84 (0.49)			
D1. I would like to take other intermediate modules in addition to my selected module		4.02 (0.63)			
D2. I approve of the block teaching approach in biostatistics		3.90 (0.70)			
D3. The course contents in each block is scientific and appropriate		3.64 (0.77)			

^a^All items were measured on a Likert 5-point agreement response scale(1 = strongly disagree; 2 = disagree; 3 = neutral; 4 = agree; 5 = strongly agree)

^b^Cronbach’s alpha for internal consistency

^c^Partial regression coefficient and its *P* value predicted the mean score change for the block group compared to the traditional group after adjusting the imbalance characteristics between two groups

The perceptions of participants toward biostatistics were depicted in Table 3. For the inherent differences in the characteristics of age, specialty, computer basics, and research experience as shown in Table 2, multivariable linear regression models were used to identify the impact of teaching approaches on the perceptions where the score of each perception section was the dependent variable, and the teaching group was an independent variable with imbalance characteristics as covariates. The group variable’s *P*-values indicated a significant difference in the perception scores between the two groups, while the partial regression coefficients indicated the mean score changes of the perceptions for the block group compared to the traditional group after adjusting the imbalance characteristics.

In general, block teaching students showed more positive perceptions as compared to traditional teaching students. Regarding the course value section, more number of block teaching students were interested in biostatistics course than the traditional teaching students (3.68 vs. 3.48, *P* < 0.01) and the course value mean score increased to 0.14 due to the block teaching approach after adjusting the imbalance characteristics between two groups. A large number of block teaching students declared that they gained the knowledge and skills of biostatistics via the curriculum (3.91 vs. 3.71, *P* < 0.01) and understood its application in their future professional career (3.48 vs. 3.28, *P* < 0.01). Although all students in both groups expressed the difficulty level of biostatistics (2.79 versus 2.88, *P =* 0.34), more block teaching students did not feel the obstacles in the learning process as compared to the traditionally teaching students (21.84% vs. 17.85%).

With respect to the course comment section, students’ perceptions significantly transformed from negative to positive after the block teaching reform (mean score of the Likert 5-point question for this section: 3.66 vs. 2.97, *P* < 0.01). For example, 46.02% of the traditionally teaching students considered the course content to be inadequate for their requirements, and 67.31% stated that the course did not provide a balance between theory and practice. However, in the block teaching group, 60.52% of the students considered the curriculum to be highly scientific and reasonable, and 74.75% considered the course content as appropriate. Also, the students provided comments and suggestions about the course. For instance, 41.08% of the students considered that the curriculum should emphasize the study design and interpretation of the statistical results. Furthermore, approximately 1/5th of them suggested that software practice lessons should be scheduled corroborating the theory lessons.

In the expectations section, block teaching students showed a high frequency in accepting suggestions beneficial to their learning. For example, many supported public lectures introducing hot statistical issues (4.25 vs. 4.02, *P* < 0.01). Additionally, 77.96% of the block teaching students approved of the teaching reform, and 82.77% were willing to attend other intermediate modules in addition to their selected module. However, a few students indicated some unreasonable issues in the curriculum structure, such as content duplication between intermediate modules (4.41%).

**Fig. 1:**
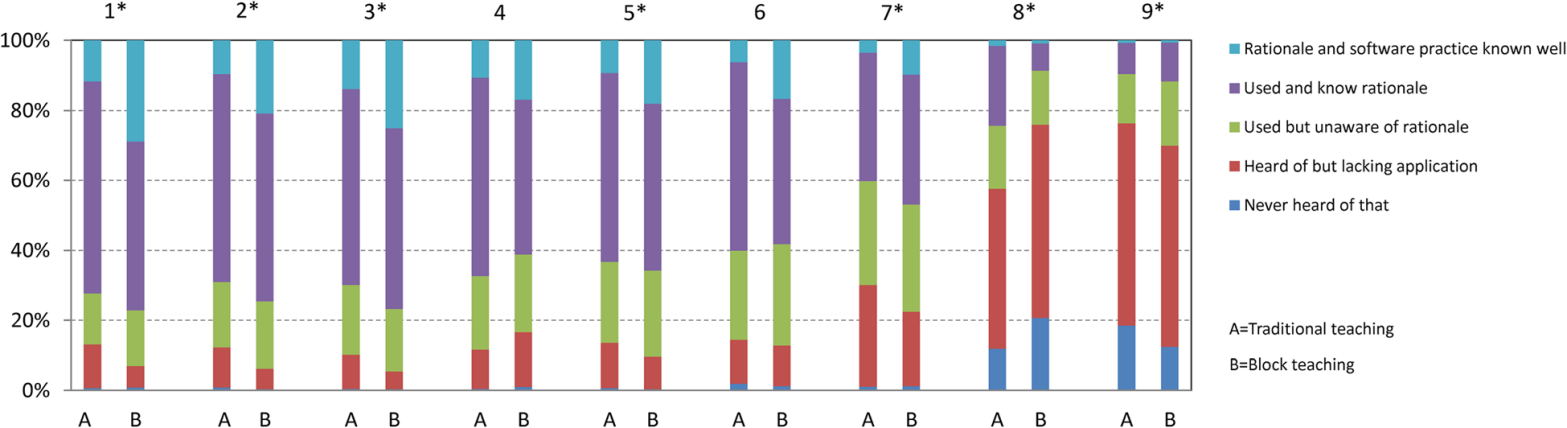
Frequencies for self-assessment ability of statistical knowledge between the traditional teaching group and block teaching group

Furthermore, the students’ self-assessment ability with academic statistical knowledge was also investigated. The responses were detailed as percentages in Fig. 1 and the statistical significance was presented with asterisk using the chi-square test. The block teaching students were rather acquainted with descriptive statistics, internal estimation, *t*-test, chi-square test, and linear regression and correlation. Compared to the traditional teaching group, almost 25% more students in the block group could apply the knowledge with rationale and software practice. However, no statistically significant difference was observed in the self-assessment ability regarding the knowledge of analysis of variance and nonparametric test between the two groups. For survival analysis and multiple linear regressions, more than half the students showed lack of application-based statistical skill, which should be addressed, and hence, comprehensible lessons should be provided in the future work.

### Factors associated with perceptions in the block group

The factors associated with the perception scores for the block teaching students with univariate analysis were presented in Table 4. No significant difference was observed in the perceptions between males and females. Students < 25 years of age were more confident about academic knowledge (3.52 vs. 3.34, *P* < 0.01) than those > 25 years, who were eager to improve their learning according to the expectation section (4.33 vs. 4.19, *P* < 0.01). For the specialty, students majoring in medical research had the most positive attitudes on course value, course comment, and block teaching approach. Moreover, students with sufficient logical thinking, mathematics, and computer basics were more positive than those with poor basics (*P* < 0.05). In addition, the research experience aided the students in obtaining maximum course value (3.58 vs. 3.38, *P* < 0.01), positive expectations (4.35 vs. 4.20, *P* < 0.01) and superior applicability on academic knowledge (3.50 vs. 3.24, *P* < 0.01).

**Table 4:** The characteristics associated with the perceptions of the block teaching students with univariate analysis

Independent variable		Value		Comment		Expectation		Reform		Ability	
		Mean(SD)	*P*-value	Mean(SD)	*P*-value	Mean(SD)	*P*-value	Mean(SD)	*P*-value	Mean(SD)	*P*-value
Sum		3.50(0.56)		3.66 (0.58)		4.29 (0.46)		3.84(0.49)		3.39 (0.63)	
Age	< 25	3.54 (0.45)	0.35	3.70 (0.48)	0.29	4.19 (0.40)	< 0.01	3.81 (0.41)	0.45	3.52(0.51)	< 0.01
	≥25	3.49 (0.54)		3.64 (0.56)		4.33 (0.43)		3.85 (0.46)		3.34 (0.60)	
Gender	Male	3.52 (0.58)	0.16	3.67 (0.59)	0.32	4.31 (0.44)	0.46	3.85 (0.50)	0.43	3.45 (0.63)	0.29
	Female	3.44 (0.53)		3.61 (0.59)		4.27 (0.48)		3.81 (0.49)		3.38 (0.58)	
Degree	Academic	3.49 (0.56)	0.46	3.66 (0.57)	0.77	4.29 (0.45)	0.91	3.86 (0.48)	0.17	3.44 (0.63)	0.01
	Professional	3.53 (0.57)		3.64 (0.61)		4.30 (0.48)		3.79 (0.49)		3.29 (0.61)	
Specialty	Clinical	3.34 (0.62)	0.01	3.46 (0.54)	0.01	3.63 (0.60)	0.72	4.27 (0.45)	0.02	3.83 (0.48)	0.84
	Research	3.56 (0.58)		3.65 (0.63)		3.70 (0.62)		4.40 (0.48)		3.84 (0.55)	
	Others	3.40 (0.68)		3.50 (0.52)		3.64 (0.61)		4.21 (0.47)		3.86 (0.40)	
Logical thinking	Poor and very poor	3.19 (0.55)	< 0.01	3.59 (0.55)	0.01	4.14 (0.38)	0.10	3.77 (0.35)	0.13	3.07 (0.53)	< 0.01
	Neutral	3.42 (0.55)		3.59 (0.59)		4.29 (0.46)		3.80 (0.49)		3.32 (0.64)	
	Good and very good	3.65 (0.54)		3.75 (0.58)		4.31 (0.47)		3.89 (0.50)		3.54 (0.59)	
Mathmetical basics	Poor and very poor	3.31 (0.53)	< 0.01	3.51 (0.56)	< 0.01	4.30 (0.45)	0.46	3.76 (0.48)	0.11	3.18 (0.66)	< 0.01
	Neutral	3.52 (0.51)		3.66 (0.60)		4.30 (0.47)		3.87 (0.48)		3.38 (0.60)	
	Good and very good	3.66 (0.67)		3.78 (0.55)		4.24 (0.46)		3.83 (0.49)		3.64 (0.58)	
Computer basics	Poor and very poor	3.30 (0.66)	< 0.01	3.55 (0.57)	0.38	4.21 (0.44)	0.18	3.81 (0.45)	0.76	3.06 (0.66)	< 0.01
	Neutral	3.53 (0.50)		3.66 (0.58)		4.30 (0.46)		3.85 (0.47)		3.46 (0.59)	
	Good and very good	3.60 (0.59)		3.72 (0.58)		4.32 (0.47)		3.84 (0.55)		3.49 (0.60)	
Research experience	None	3.38 (0.54)	< 0.01	3.65 (0.55)	0.87	4.20 (0.48)	< 0.01	3.80 (0.46)	0.16	3.24 (0.60)	< 0.01
	Yes	3.58 (0.56)		3.66 (0.61)		4.35 (0.44)		3.86 (0.50)		3.50 (0.62)	

### Correlations between perceptions and academic achievement

At the end of the semester, postgraduates took a final exam that was objective and quantitative. Therefore, the examination score was applied as the academic achievement to evaluate the level of knowledge mastered in this course. The mean scores on the examination were 69.49 with a standard deviation of 8.79 for the block cohort and 65.32 with a standard deviation of 11.47 for the traditional cohort (hundred-mark system).

Assuming that the five sections in the questionnaire were five latent perception constructs reflected by their respective items, a confirmatory factor analysis (CFA) [30] using SEM [31] was conducted to assess the linear association between perception and examination achievement in the block teaching group. Maximum likelihood estimation was utilized to estimate the parameters, and several indices from a chi-square test were considered to assess the model fit [32]. The items in the questionnaire were statistically significant (*P* < 0.05) in the resulting SEM, of which, the chi-square test was statistically significant (${\chi}_{267}^2=654.905$, *P* < 0.0001), the comparative fit index (CFI) was (0.968) > 0.90, the root-mean-square error of approximation (RMSEA) was (0.055) < 0.06, and the degree of freedom ratio was (2.45) < 3.0.

**Fig. 2:**
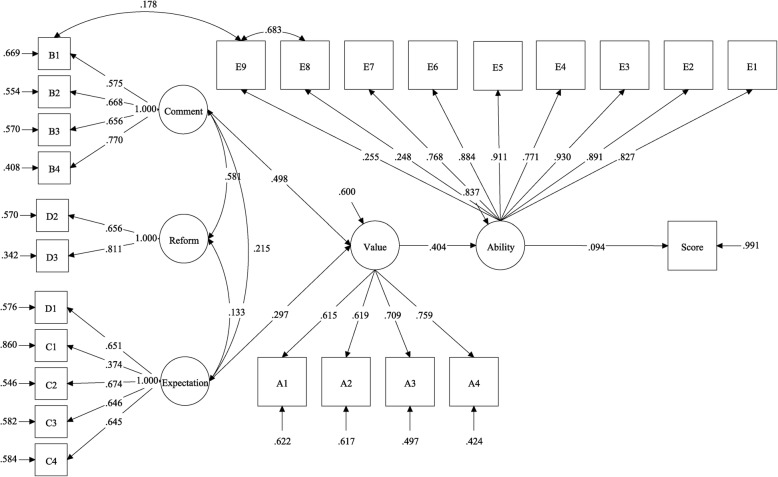
Parameter estimates (standard regression coefficients) of the structure equation model for the linear relationship between students’ perceptions and academic achievement on the biostatistics course

These results were detailed in Fig. 2. As indicated, the course comment, block teaching reform, and course expectation were positively related to one another (standardized regression coefficient *β* = 0.581,*β* = 0.215, and *β* = 0.215). Moreover, the course comment and expectation were positively affected by course value (*β* = 0.498 and *β* = 0.297, respectively). Then, the course value positively affected the self-assessment ability level of statistical knowledge (*β* = 0.404). Finally, the latent ability variable positively affected the achievement score (*β* = 0.094). For the explanatory power of the SEM, the course comment and expectation accounted for 40% of the variance in the course value. Value perceptions accounted for 16.30% of the variance related to the self-assessment ability variable. However, the ability resulted in only 9.4% of the variance in the exam score, which indicated the presence of other latent factors that might be linked to the achievement score.

## Discussion

Medical students often complain that biostatistics is difficult and confusing [19, 33], as it required to acquire an in-depth knowledge of mathematics and calculation. Herein, we conducted a block curriculum design on a biostatistics course. This study identified that the block design could reduce the students’ negative perceptions while learning biostatistics, thereby further improving their course achievement.

Compared to the traditional teaching approach, the innovations of the block teaching approach lie in the curriculum design and teaching style. In the block curriculum, the syllabus and constructions were redesigned to consist of three different difficulty levels and research directions. The basic block was concerned with introductory statistics that are relatively simple and critical in shaping students’ beliefs and attitudes [34]. Along with the recommendations by Evans [35] and Mintz [36], mathematics and complicated formulae were reduced, and the teaching propagated real examples [9, 37, 38]. For the intermediate block, highlighting the students’ professional needs and providing selective directions might encourage the students to be open and positive towards understanding and applying statistics. The advanced block combined new technology and pragmatic health issues such that statistical thinking could be embedded in interpretation and critical judgment. Moreover, we maximized the strategy to provide blended learning styles using a variety of media and materials, such as lectures, software, and videos, especially, by increasing the small-group problem-solving component in the teaching, wherein students explore the statistical concepts in great detail; this phenomenon was in line with the recommendations of several researchers. The survey of two cohorts with a relatively large sample showed that postgraduates experiencing block teaching tended to hold positive perceptions and improved self-assessment ability in biostatistics. More than half of them planned for further improvement in their statistical skills for the academic career. Therefore, the block teaching reform might obtain the desired learning outcomes.

Findings from SEM also provided some support for the positive association between perceptions and course achievements [18, 39]. Specifically, course value is a positive predictor of self-assessment ability of biostatistics and course achievement, which was in agreement with the control-value theory [17, 18]. The results suggested that students who considered the course to be interesting, significant, and useful were more likely to enjoy the knowledge gained. Also, those who were confident about learning the course material were less likely to experience course-related anxiety [16]. Enjoyment, confidence, and positive emotions might impact the achievement outcomes. In this study, although the overall effects of the SEM accounted for only 9.4% variation in the exam score, the medium effect sizes are in line with the limited empirical evidence [18, 20]. These results suggest that statistical educators should consider and explicitly address the students’ course-related perceptions, which play a critical role and are moderately linked to their academic achievement.

Although several scales measuring perceptions toward statistics have been developed, such as the Survey of Attitudes Toward Statistics (SATS) [25] and the Attitudes Toward Statistics scale (ATS) [24], we developed a questionnaire to survey the medical students’ perceptions. In a previous study [19], we validated the importance of attitudes toward statistics in postgraduate medical training using the SATS scale. In this study, we aimed to assess the contribution of block curriculum design in reducing the students’ negative perceptions. Also, we expected additional suggestions relating the block teaching reform from students to further optimize the curriculum design. Therefore, the expectation and reform sections towards the block design were restructured, and the ability section incorporated nine statistical academic questions to assess the mastery of knowledge. The survey showed acceptable validity and reliability of the instrument. With some semi-open questions, the participants in the survey offered crucial feedback in terms of our teaching strategy. For example, they wanted to understand the latest technology concerning biostatistics, such as diagnostic tests, meta-analysis, clinical trial design, and real research. These findings provided valuable suggestions to biostatistics educators for improving the course content, teaching methods, and block curriculum schemes.

### Limitations

Compared to the traditional group, more students in the block group had prior research experience, and thus, may grasp some basic ability of statistical analysis and recognize the importance of biostatistics, which might promote the students’ positive perceptions toward biostatistics. A stratification analysis in the appendix (Additional file 2) showed that the block teaching students exhibited positive perceptions irrespective of the research experience. Although statistical methods were applied to adjust the bias, these could not account for all the confounders between the groups.

Furthermore, an author-designed questionnaire was employed for measuring the perceptions rather than using the existing instruments; for example, SATS. The survey results could not be compared with other studies using standard scales.

Also, the perceptions were collected using the 5-point Likert style, and the absolute differences in the perception scores were numerically small and their practical significance of the small absolute difference may be limited.

## Conclusion

Statistical education is not just to impart knowledge by a didactic approach but also to provide students with a comprehensive statistical thinking and self-perceived confidence. The current innovation of block curriculum design improved the postgraduates’ positive perceptions and may exert a positive role in improving the postgraduates’ achievement of learning biostatistics. However, practical technology in the curriculum design of biostatistics should be under intensive focus. An enhanced selective curriculum covered different difficulty levels that could cope with the students’ professional needs. Diverse statistical design and analysis methods should be emphasized specifically for different research directions. These measures may improve the students’ real-life data analysis and ease their fear and anxiety of learning about biostatistics. Moreover, effective teaching styles and interventions are also welcomed to improve the students’ positive perceptions for developing their self-directed learning and life-long learning for a future career.

## Additional files

Additional file 1: The self-administered questionnaire concerning perceptions towards biostatistics (DOCX 24 kb)

Additional file 2: Stratification analysis of perceptions towards biostatistics with or without research experience (DOCX 21 kb)

## Abbreviations

ANOVA: Analysis of variance
ATS: Attitudes Toward Statistics scale
SATS: Survey of Attitudes Toward Statistics
SEM: Structural equation modeling

## References

[1] Bryant JH. Education tomorrow’s doctors. In: World health forum: WHO; 1993. p. 217–30. 8397729

[2] Hunponu-Wusu OO (1977). The need for medical statistics in the training of health personnel. Med Educ.

[3] Garfield J (2003). Assessing statistical reasoning. Stat Educ Res J.

[4] Brimacombe MB (2014). Biostatistical and medical statistics graduate education. Bmc Med Educ.

[5] Cobb GW, Moore DS (1997). Mathematics, statistics, and teaching. Am Math Mon.

[6] Council G: Tomorrow ʼs doctors - Outcomes and standards for undergraduate medical education London*:* General Medical Council 2009.

[7] Garfield J (1995). How students learn statistics. Int Stat Rev.

[8] Martin BJ (2004). Teaching statistics to medical students using problem-based learning: the Australian experience. Bmc Med Educ.

[9] Freeman JV, Collier S, Staniforth D, Smith KJ (2008). Innovations in curriculum design: a multi-disciplinary approach to teaching statistics to undergraduate medical students. Bmc Med Educ.

[10] Luo L, Cheng X, Wang S, Zhang J, Zhu W, Yang J, Liu P (2017). Blended learning with Moodle in medical statistics: an assessment of knowledge, attitudes and practices relating to e-learning. Bmc Med Educ.

[11] Marantz PR, Burton W, Steinergrossman P (2003). Using the case-discussion method to teach epidemiology and biostatistics. Acad Med.

[12] Gal I, Ginsburg L: The role of beliefs and attitudes in learning statistics: towards an assessment framework**.** J Stat Educ [on-line serial 1994.

[13] Garfield J, Hogg B, Schau C, Whittinghill D. First courses in statistical science: the status of educational reform efforts. J Stat Educ. 2001, 10

[14] Gal I, Ginsburg L, Schau C: Monitoring attitudes and beliefs in statistics education**.** Igal & Jbgarfield 1997.

[15] Eccles JS, Wigfield A (2002). Motivational beliefs, values, and goals. Annu Rev Psychol.

[16] Bandura A, Schunk DH (1981). Cultivating competence, self-efficacy, and intrinsic interest through proximal self-motivation. J Perso Soc Psychol.

[17] Pekrun R, Goetz T, Titz W, Perry RP (2002). Academic emotions in Students’ self-regulated learning and achievement: a program of qualitative and quantitative research. Educ Psychol.

[18] Pekrun R, Elliot AJ, Maier MA: Achievement goals and achievement emotions: testing a model of their joint relations with academic performance**.** J Educ Psychol 2009, 101**:**págs. 115–135.

[19] Zhang Y, Lei S, Rui W, Zhao Q, Li C, Xu Y, Su H. Attitudes toward statistics in medical postgraduates: measuring, evaluating and monitoring. Bmc Med Educ. 2011, 12:1–8. 10.1186/1472-6920-12-117 PMC3533942 23173770

[20] Khan N, Mumtaz Y (2009). Attitude of teaching faculty towards statistics at a medical university in Karachi, Pakistan. J Ayub Med College Abbottabad Jamc.

[21] Artino AR, Rochelle JS, La DSJ (2010). Second-year medical students’ motivational beliefs, emotions, and achievement. Med Educ.

[22] Robbins SB, Lauver K, Le H, Davis D, Langley R, Carlstrom A (2004). Do psychosocial and study skill factors predict college outcomes? A meta-analysis. Psychol Bull.

[23] Emmioglu E, Capa-Aydin Y (2012). Attitudes and achievement in statistics: a meta-analysis study. Stat Educ Res J.

[24] Waters LK, Martelli TA, Zakrajsek T, Popovich PM (1988). Attitudes toward statistics: an evaluation of multiple measures. Educ Psychol Measur.

[25] Cashin SE, Elmore PB (2005). The survey of attitudes toward statistics scale: a construct validity study. Educ Psychol Meas.

[26] Wise SL (1985). The development and validation of a scale measuring attitudes toward statistics. Educ Psychol Measur.

[27] Schau C, Others A (1995). The development and validation of the survey of Antitudes toward statistics. Educ Psychol Measur.

[28] Saris W, Den Ronden J, Satorra A, Cuttance P, Ecob J. Testing structural equation models. In: Structural modeling by example: Applications in educational, sociological, and behavioral research 1987. p. 202–20.

[29] Bland JM, Altman DG (1997). Statistics notes: Cronbach’s alpha. BMJ.

[30] Brown, TA. Confirmatory factor analysis for applied research. New York: Guilford Press;2006.

[31] Kline, RB. Principles and practice of structural equation modeling (3rd ed.). New York: Guilford Press;2010.

[32] Hu LT, Bentler PM (1999). Cutoff criteria for fit indexes in covariance structure Anaysis: conventional criteria versus new alternatives. Struct Equ Model Multidiscip J.

[33] Hannigan A, Hegarty AC, Mcgrath D (2014). Attitudes towards statistics of graduate entry medical students: the role of prior learning experiences. Bmc Med Educ.

[34] Roiter K, Petocz P (1996). Introductory statistics courses-a new way of thinking. J Stat Educ.

[35] Evans SJ (1990). Statistics for medical students in the 1990’s: how should we approach the future?. Stat Med.

[36] Mintz E, Ostbye T (1992). Teaching statistics to health professionals: the legal analogy. Med Teacher.

[37] Moore DS (1993). The place of video in new styles of teaching and learning statistics. Am Stat.

[38] Nooriafshar M (2005). A comparison of learning preferences and perceptions of students for statistics concepts and techniques. Int J Math Teach Learn.

[39] Pekrun R (2006). The control-value theory of achievement emotions: assumptions, corollaries, and implications for educational research and practice. Educ Psychol Rev.

