# Supplementary material for: Assessment of a block curriculum design on medical postgraduates’ perception towards biostatistics: a cohort study
Source: BMC Med Educ. 2018 Jun 19;18:144. doi: 10.1186/s12909-018-1232-0 (PMC6006669; doi:10.1186/s12909-018-1232-0)
Supplement: Supplementary file 1 — The self-administered questionnaire concerning perceptions towards biostatistics (DOCX 24 kb) [file 12909_2018_1232_MOESM1_ESM.docx]

S1 The self-administered questionnaire concerning perceptions towards biostatistics

The Section D “Reform” was only provided for the postgraduates receiving block curriculum design.

**Perceptions toward biostatistics in medical postgraduate education**

This is a self-administered questionnaire concerning your perception and attitude towards biostatistics. No name was required to be registered in the questionnaire. All the questions you answered were assured without any impact on your academic achievement or your future learning. Please complete it truthfully. Thank you.

***Demography***

| **Age** |  |  |  |  |  |
| --- | --- | --- | --- | --- | --- |
| **Gender** | Male⬜ | Female⬜ |  |  |  |
| **Degree** | Academic ⬜ | Professional ⬜ |  |  |  |
| **Specialty** | Clinical⬜ | Research ⬜ | Others ⬜ |  |  |
| **Logical thinking** | Very poor ⬜ | Poor ⬜ | Neutral ⬜ | Good ⬜ | Very good ⬜ |
| **Mathematics basis** | Very poor ⬜ | Poor ⬜ | Neutral ⬜ | Good ⬜ | Very good ⬜ |
| **Computer basis** | Very poor ⬜ | Poor ⬜ | Neutral ⬜ | Good ⬜ | Very good ⬜ |
| **Research** **experience** | Yes ⬜ | No ⬜ |  |  |  |

***Perception***

For questions 1-12 please circle the number best described your preference, which is represented as 1=Strongly disagree, 2=Disagree, 3=Neutral, 4=agree, and 5=Strongly agree.

**Section A: Value**

A1. I am interested in biostatistics

1 2 3 4 5

A2. I have no obstacle in learning biostatistics

1 2 3 4 5

A3. I gained useful statistical knowledge and skills by taking this course

1 2 3 4 5

A4. I know how to use the statistical knowledge in my professional career

1 2 3 4 5

**Section B: Comment**

B1. This course introduced adequate knowledge to satisfy my practical career goals

1 2 3 4 5

B2. The course framework is scientific and reasonable

1 2 3 4 5

If opposed, please indicate the drawbacks (multiple-choice question)

1. The sequence of chapters is unreasonable
2. Too much knowledge is required to learn in short duration
3. Course content is not practical for professional career
4. Software practice is inadequate
5. Others

B3. The theory and practice are well combined in the teaching form

1 2 3 4 5

B4. The course content is appropriate

1 2 3 4 5

If opposed, please suggest which content should be emphasized?

1. Study design
2. Mathematics and calculation
3. Software practice
4. Interpretation if the results
5. Others

**Section C: Expectation**

C1. Need more software practice lessons

1 2 3 4 5

C2. Need more practical workshop for research design and analysis in real study

1 2 3 4 5

C3. Provide comprehensive guide handbook for biostatistics like other medical subject

1 2 3 4 5

C4. Give public lecture introducing the latest and hot issues relating biostatistics

1 2 3 4 5

If agreed, what do you want to learn more in the area of biostatistics? (multiple-choice question)

| A. Toxicologic study | B. Genetic study |
| --- | --- |
| C. Bioinformatics | D. Diagnostic test |
| E. (New drug) Clinical trial | F. Investigation |
| G. Screening test | H. Meta-analysis |
| I. Surveillance | J. Decision analysis |
| k. Others | |

**Section D: Reform**

D1. I would like to learn the other module course in addition to my selected module

1 2 3 4 5

D2. I approved the block education on biostatistics course

1 2 3 4 5

D3. The course content of block curriculum design is scientific and appropriate

1 2 3 4 5

If opposed, please indicate the drawbacks (multiple-choice question)

1. The course content is not corresponding with the name of the corresponding module
2. The course contents are similar between different modules
3. The course contents can not meet the needs in practice
4. The course contents is difficult in the advanced modularized sections
5. Others

**Section E: Ability**

For academic knowledges 1-9 please circle the number best described your acquaintance level, which is represented as 1=never heard, 2= heard but lacked application, 3= applied but unaware of rationale, 4= applied with rationale, and 5= applied with rationale and software practice.

E1. Descriptive statistics

1 2 3 4 5

E2. Interval estimation

1 2 3 4 5

E3. *t*-test

1 2 3 4 5

E4. Analysis of variance

1 2 3 4 5

E5. Chi-square test

1 2 3 4 5

E6. Nonparametric test

1 2 3 4 5

E7. Linear regression and correlation

1 2 3 4 5

E8. Survival analysis

1 2 3 4 5

E9. Multiple linear regression

1 2 3 4 5
