# Supplementary material for: Assessment of a block curriculum design on medical postgraduates’ perception towards biostatistics: a cohort study
Source: BMC Med Educ. 2018 Jun 19;18:144. doi: 10.1186/s12909-018-1232-0 (PMC6006669; doi:10.1186/s12909-018-1232-0)
Supplement: Supplementary file 2 — Stratification analysis of perceptions towards biostatistics with or without research experience (DOCX 21 kb) [file 12909_2018_1232_MOESM2_ESM.docx]

S2 Stratification analysis of perceptions towards biostatistics with the research experience have or not

Table s1 Stratification analysis of perceptions towards biostatistics

| **Survey sections** | **Mean Score (SD) ^a^** | | **Mean Score**  **Difference**  **(B-T)** | **Partial regreesion**  **Coefficient**  **(95%CI)^b^** | ***P* value^c^** |
| --- | --- | --- | --- | --- | --- |
|  | **Traditional teaching** | **Block teaching** |  |  |  |
| Have no research experience |  |  |  |  |  |
| **Section A: Value** | 3.36(0.51) | 3.38(0.54) | 0.02 | 0.08 (-0.02,0.18) | 0.12 |
| **Section B: Comment** | 2.95(0.54) | 3.65(0.55) | 0.70 | 0.65 (0.55,0.75) | <0.01 |
| **Section C: Expectation** | 4.00(0.47) | 4.20(0.48) | 0.20 | 0.19 (0.10,0.28) | <0.01 |
| Have research experience |  |  |  |  |  |
| **Section A: Value** | 3.39(0.48) | 3.58(0.56) | 0.19 | 0.19 (0.09,0.29) | <0.01 |
| **Section B: Comment** | 3.00(0.56) | 3.66(0.61) | 0.66 | 0.67 (0.56,0.78) | <0.01 |
| **Section C: Expectation** | 4.02(0.47) | 4.35(0.44) | 0.33 | 0.31 (0.23,0.39) | <0.01 |

a: All items in each section were measured on a Likert 5-point agreement response scale(1=strongly disagree; 2= disagree; 3=neutral; 4=agree; 5=strongly agree)

b: Partial regression coefficient predicted the mean score change for the block group compared to the traditional group after adjusting the imbalance characteristics between two groups

c: The *P* value of the teaching group variable in the multivariable linear regression model
